# A novel endpoint for liver surgery predicts surgery-related mortality: external validation in a surgical cohort with intrahepatic cholangiocarcinoma

**DOI:** 10.3389/fsurg.2026.1835401

**Published:** 2026-05-15

**Authors:** Constantin Scholz, Evangelos Tagkalos, Franziska Renger, Monia Passalacqua, Lukas Müller, Maximilian Moos, Lisa Katharina Gröger, Jens Mittler, Janine Baumgart, Tobias Huber, Friedrich Foerster, Arndt Weinmann, Fabian Bartsch, Hauke Lang

**Affiliations:** 1 Department of General, Visceral and Transplant Surgery, University Medical Center Mainz, Mainz, Germany; 2 Department of Diagnostic and Interventional Radiology, University Medical Center Mainz, Mainz, Germany; 3 Department of Internal Medicine, Gastroenterology and Hepatology, University Medical Center Mainz, Mainz, Germany; 4 Department of General and Visceral Surgery, Hospital “Barmherzige Brüder” Trier, Trier, Germany

**Keywords:** clinical study, composite endpoint, endpoints, hepatectomy, intrahepatic cholangiocarcinoma, liver surgery, postoperative complications, validation study

## Abstract

**Background:**

The composite endpoint for liver surgery (CELS) was developed to predict surgery-related mortality and to improve the design of clinical trials in liver surgery. However, external validation beyond the initial development cohort remains limited. This study aimed to assess the validity and reliability of CELS in a distinct patient population undergoing liver resection for intrahepatic cholangiocarcinoma.

**Methods:**

The primary objective was to assess the association between CELS and surgery-related mortality. Secondary objectives included 30-day mortality, length of hospital stay (LOS), overall survival, and recurrence-free survival. Predictive performance was evaluated using sensitivity, specificity, accuracy, and receiver operating characteristic curve analyses.

**Results:**

A total of 227 patients were included in the analysis (CELS-positive: *n* = 87; CELS-negative: *n* = 140). A total of 58 minor, 62 major, and 107 extended resections were performed. The 90-day mortality rate (surgery-related mortality) was 8.9% in the overall cohort and 9.6% in the validation cohort. The CELS-positive group was more frequently affected by surgery-related mortality compared to the CELS-negative group (20.7% vs. 3.6%, *p* *<* 0.001). CELS demonstrated a sensitivity of 81.8%, a specificity of 66.3%, and an overall accuracy of 67.8% in predicting surgery-related death. The discriminatory ability of CELS was moderate, with an area under the receiver operating characteristic (ROC) curve of 0.74.

**Conclusions:**

CELS demonstrated moderate predictive ability for surgery-related mortality following liver resection for intrahepatic cholangiocarcinoma.

## Introduction

Selecting appropriate endpoints to assess outcomes of hepatobiliary surgery in clinical trials remains a significant challenge. While various specific endpoints, such as reoperation rates, length of hospital stay, and postoperative complications, are commonly used to assess surgical outcomes, they often limit comparability across studies. Moreover, these single-component endpoints are less frequent, requiring larger sample sizes, reducing statistical efficiency, and increasing the financial burden to maintain adequate statistical power.

Survival-based endpoints, although clinically meaningful, become increasingly impractical as their event rates decline with therapeutic advances. Trials relying solely on overall survival (OS) as a primary endpoint may require extended follow-up, leading to increased financial expenditure and extended study durations. A proposed solution to these issues is the use of surrogate or composite endpoints. These approaches can increase event rates, enhance statistical efficiency, and improve cost-effectiveness by reducing the required sample size.

Composite endpoints, by definition, combine multiple individual outcomes into a single measure, thereby increasing the event rate. Nevertheless, they are subject to an inherent risk of internal competition among component events, which may compromise interpretability (1). Therefore, any newly proposed composite endpoint must meet rigorous standards and undergo thorough validation before adoption in prospective randomized trials.

In this context, Kawashima and Pawlik et al. proposed a composite endpoint for liver surgery—termed the composite endpoint for liver surgery (CELS)—which includes posthepatectomy liver failure, bile leakage, posthepatectomy hemorrhage, and intraoperative blood loss exceeding 2 L as predictors for surgery-related mortality (2). The CELS was developed using both internal and external patient cohorts and is intended to capture the multifaceted nature of surgical outcomes following liver resection, thereby facilitating trials in this field. According to the original validation study, use of the CELS could reduce required sample sizes in clinical trials by up to 90%.

Despite these promising findings, further validation of the CELS across diverse institutions and healthcare systems is necessary to assess its generalizability and ensure its reliability and validity as a standard clinical trial endpoint. In the present study, we aimed to validate the predictive capacity of the CELS in a retrospective cohort of patients with intrahepatic cholangiocarcinoma (iCCA) undergoing liver resection; a relatively small sample size was intentionally selected to test the limits of the CELS. Furthermore, we evaluated its potential utility as a surrogate endpoint for long-term oncological outcomes, including overall survival and recurrence-free survival (RFS).

## Methods

We conducted a validation study to investigate the predictive ability of a new CELS for surgery-related death (SRD) at the University Medical Center Mainz. The study was performed as an exploratory analysis including patients with intrahepatic cholangiocarcinoma undergoing curative liver resection. All patients who underwent curative liver resection between 2008 and 2024 were evaluated for study inclusion. Those with irresectable disease were excluded. Follow-up data were updated annually through contact with outpatient oncologists or family physicians. The study was reported in accordance with the TRIPOD statement (3).

### Endpoints and predictors

The primary objective of this study was to validate the CELS as a surrogate for surgery-related death. Surgery-related death was defined as death within 90 days after surgery. CELS is a composite endpoint consisting of posthepatectomy liver failure (PHLF; grades A–C), posthepatectomy bile leakage (grades A–C), posthepatectomy hemorrhage (PHH; grades A–C) as defined by the International Study Group of Liver Surgery (ISGLS), and blood loss exceeding 2 L (BL) during the index surgery (3–6).

Secondary endpoints included 30-day mortality, length of hospital stay (days), overall survival, and recurrence-free survival. Overall survival was defined as the time from index surgery to death from any cause. Recurrence-free survival was defined as the time from index surgery to the first appearance of disease recurrence.

Further, we evaluated the survival outcomes stratified according to resection type. Minor resections were defined as resection involving fewer than three segments, major resections as those involving—three to four segments. Extended resections were defined as resections involving five or more segments, central hepatectomy, associating liver partition and portal vein ligation, or any resection (independent from segmental extension) combined with additional vascular or extrahepatic visceral resection.

### Statistical analysis

Data were obtained from a large prospective institutional database including more than 351 surgical patients with intrahepatic cholangiocarcinoma. As this was a retrospective analysis, no sample size calculation was performed. Cases with missing data for one or more variables were excluded, and only complete cases were included in the statistical analysis (Supplementary Table S1a).

Continuous variables were presented as mean with standard deviation, and categorical variables were reported as absolute and relative frequencies. Differences between the CELS-positive (CELS+) and CELS-negative (CELS−) cohorts were analyzed using the chi-square or Fisher’s exact test for categorical variables and Welch's *t*-test for continuous variables, as appropriate. Given the robustness of Welch's *t*-test to outliers, testing for normality was not required. The predictive ability of the CELS for primary and secondary endpoints was evaluated using diagnostic parameters such as sensitivity, specificity, negative predictive value (NPV), positive predictive value (PPV), and overall accuracy. Receiver operating curve analysis was used to assess the discriminatory ability of CELS, as measured by the area under the curve (AUC). Time-to-event analyses for overall survival and recurrence-free survival were conducted using Kaplan–Meier analysis.

All statistical tests were reported using 95% confidence intervals. Data analysis was conducted using IBM SPSS (version 29.0.0). The plots were visualized using GraphPad Prism (version 10.4.2.) and R Studio (version 4.5.0).

### Ethical considerations

All enrolled patients provided informed consent, authorizing the collection of data and its anonymous use for potential scientific analyses. In compliance with the stipulations outlined in the state hospital law (§36 and §37) of the federal state and the directives of the independent ethics committee of Rhineland-Palatinate, formal ethical approval was not required for this study. The study was performed in accordance with the Declaration of Helsinki (as revised in 2013).

## Results

### Patient characteristics

**Figure 1:**
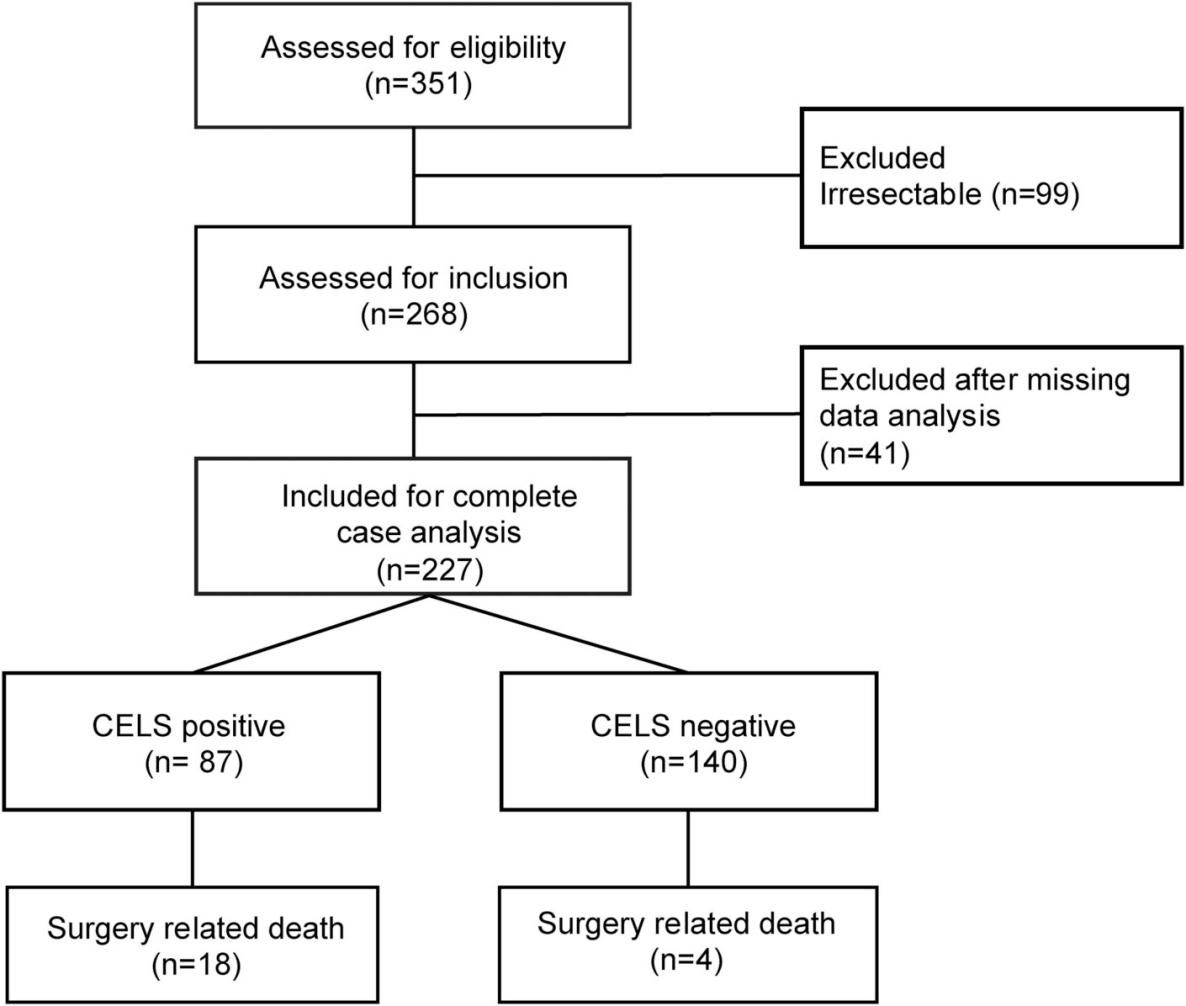
Flowchart demonstrating the stepwise inclusion process.

We screened patients with intrahepatic cholangiocarcinoma undergoing curative resection between October 2007 and January 2025 from an institutional database. After conducting a stepwise inclusion process, 227 patients were included in the final analysis (Figure 1).

A total of 48.9% of the patients were men, and 51.1% were women. Sex was equally distributed depending on CELS (CELS+: *n* = 47 male, *n* = 40 female; CELS−: *n* = 64 male, *n* = 76 female; *p* = 0.233). The mean age was 64.6 years (CELS+: 64.4 years vs. CELS−: 64.8 years; *p* = 0.781) (Table 1).

**Table 1:** Baseline characteristics.

Baseline characteristics	External validation cohort
*n* (%)	227 (100)
Sex, *n* (%)	
Male	111 (48.9)
Female	116 (51.1)
Age in years mean (SD)	64.6 (±11.6)
Height in cm mean (SD)	171.6 (±9.3)
Weight in kg mean (SD)	79.2 (±16.4)
ASA,^a^ *n* (%)	
I	3 (1.3)
II	105 (46.3)
III	113 (49.8)
IV	6 (2.6)
Procedure, *n* (%)	
Minor hepatectomy	58 (25.6)
Major hepatectomy	62 (27.3)
Extended hepatectomy	107 (47.1)
CD >3a,^b^ *n (%)*	73 (32.2)
CCI,^c^ mean (SD)	22.5 (±30.07)
Ca 19–9 U/L, mean (SD)	2,071.3 (±10,361.7)
LOS^d^ in days mean (SD)	19.2 (± 15.3)
>75th percentile LOS, *n (%)*	56 (24.7)
Surgery-related death, *n (%)*	22 (9.6)
CELS+,^e^ *n (%)*	87 (38.3)
Blood loss in mL mean (SD)	926.6 (±1,139.7)
Blood loss > 2 L, *n (%)*	21 (9.3)
Bile leakage—ISGLS,^f^ *n* (%)	
A	27 (11.9)
B	19 (8.4)
C	12 (5.3)
PHLF^h^—ISGLS, *n* (%)	
A	9 (4.0)
B	3 (1.3)
C	10 (4.4)
PHH^g^—ISGLS, *n* (%)	
A	4 (1.8)
B	10 (4.4)
C	10 (4.4)

a American Society of Anaesthesiologists.

b Clavien–Dindo grade exceeding 3a.

c Comprehensive complication index.

d Length of hospital stay.

e Positive composite endpoint for liver surgery.

f International Study Group for Liver Surgery.

g Posthepatectomy hemorrhage.

h Posthepatectomy liver failure.

### Surgery-related mortality

**Figure 2:**
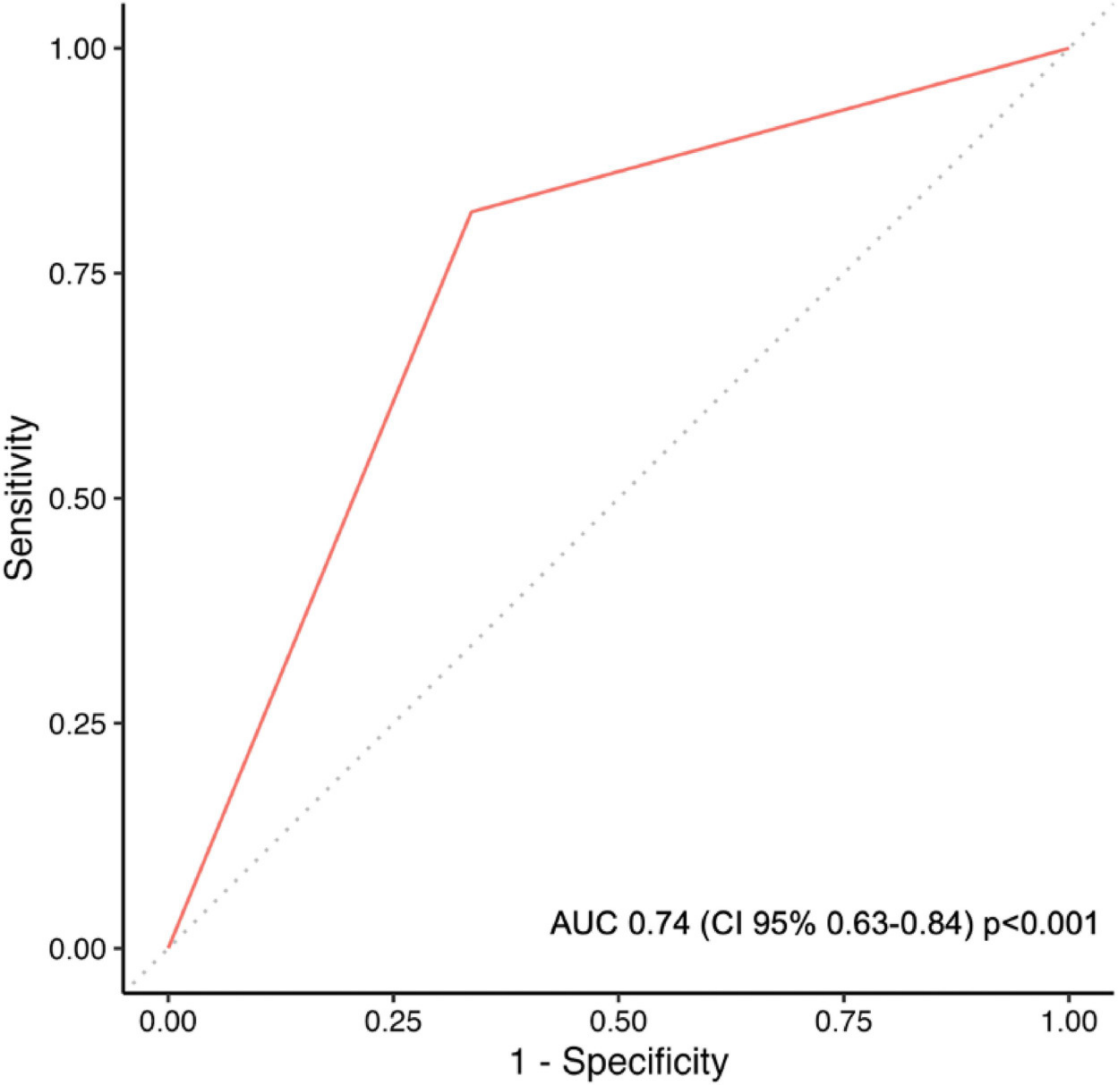
Receiver operating characteristic curve analysis demonstrating the ability of CELS to discriminate between surgery-related death and non-surgery-related death.

In total, 22 of 227 patients (9.6%) in the CELS cohort and 24 of 268 patients (8.9%) in the overall cohort experienced SRD (Table 2). The SRD rate was 20.7% (*n* = 18) in the CELS+ group and 3.6% (*n* = 4) in the CELS− group (*p* < 0.001). The predictive performance of CELS for SRD showed a sensitivity of 81.8%, a specificity of 66.3%, and an overall accuracy of 67.8%. The PPV was 20.7%, whereas the NPV was 97.1%. ROC analysis for the evaluation of the prediction model demonstrated moderate discriminatory ability (AUC, 0.74; *p* < 0.001) (Figure 2).

**Table 2:** Survival outcomes.

Resections	Total	SRD^a^	1-year survival	3-year survival	5-year survival
Overall cohort, *n (%)*	268 (100)	24 (8.9)	181 (67.5)	112 (41.8)	35 (13.1)
MiH,^b^ *n* (%)	77 (100)	0 (0)	54 (70.1)	26 (33.8)	16 (20.8)
MaH,^c^ *n* (%)	84 (100)	6 (8.1)	53 (63.1)	22 (26.2)	6 (7.1)
eMaH,^d^ *n* (%)	107 (100)	18 (14.2)	74 (69.2)	28 (35.5)	13 (12.6)
CELS cohort, *n* (%)	227 (100)	22 (9.6)	109 (48.0)	64 (28.2)	29 (12.8)
MiH, *n* (%)	58 (100)	0 (0)	49 (84.5)	25 (43.1)	15 (25.9)
MaH, *n* (%)	62 (100)	6 (9.6)	35 (56.4)	15 (56.5)	3 (4.8)
eMaH, *n* (%)	107 (100)	16 (15.0)	67 (62.6)	24 (22.4)	11 (10.3)

a Surgery-related mortality.

b Minor hepatectomy.

c Major hepatectomy.

d Extended major hepatectomy.

### Length of hospital stay

**Figure 3:**
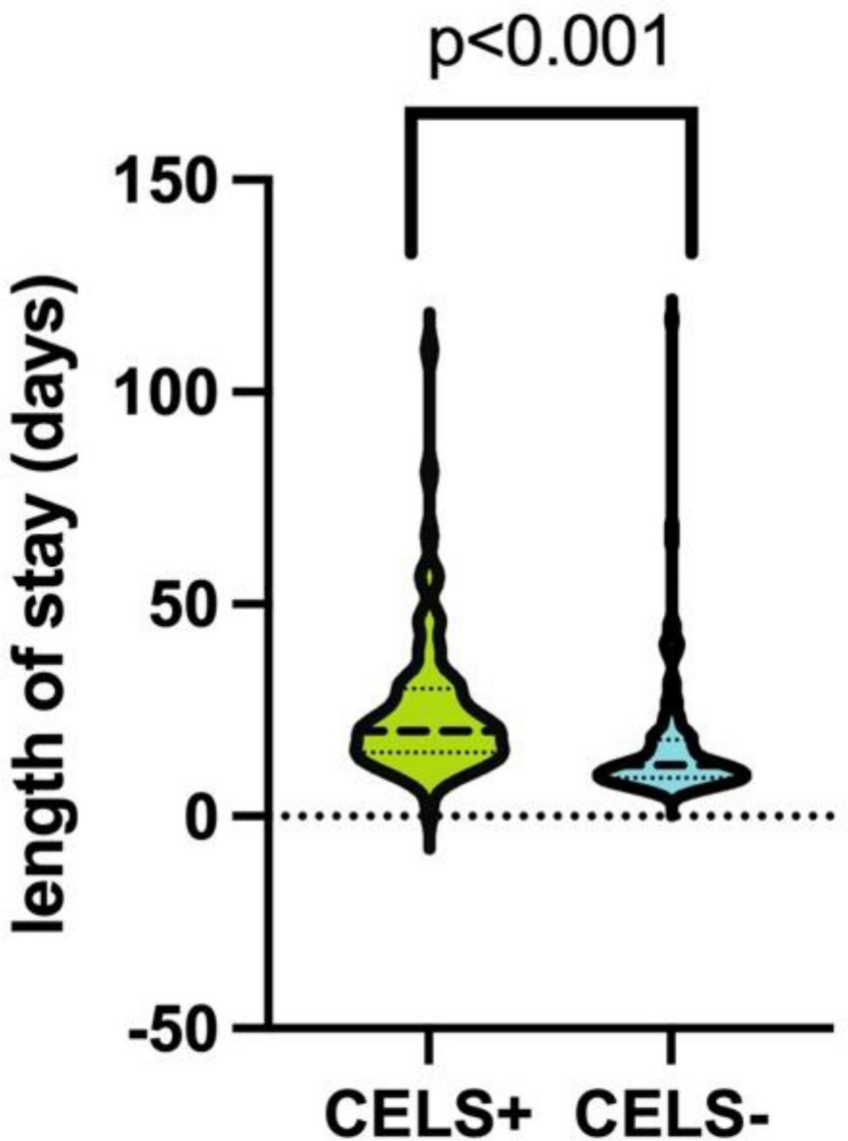
Violin plot demonstrating the length of hospital stay (LOS) in the CELS+ group (mean 25.0 days) and the CELS− group (16.0 days) *p* < 0.001.

The mean length of hospital stay (LOS) was significantly longer in the CELS+ group compared with the CELS− group (25.02 vs. 16.07 days; 95% CI: −13.24 to −4.63; *p* < 0.001) (Figure 3). In accordance with the methodology described by Pawlik et al., the 75th percentile was used as the threshold to define prolonged hospital stay. In this cohort, the 75th percentile cutoff was 22 days. CELS demonstrated a sensitivity of 62.7%, a specificity of 71.6%, and an overall accuracy of 69.2% for predicting prolonged hospital stay. The NPV was 84.1%, and the PPV was 44.6%. ROC curve analysis yielded an AUC of 0.67 (95% CI: 0.58–0.78; *p* < 0.001).

### Long-term survival and oncological outcomes

**Figure 4:**
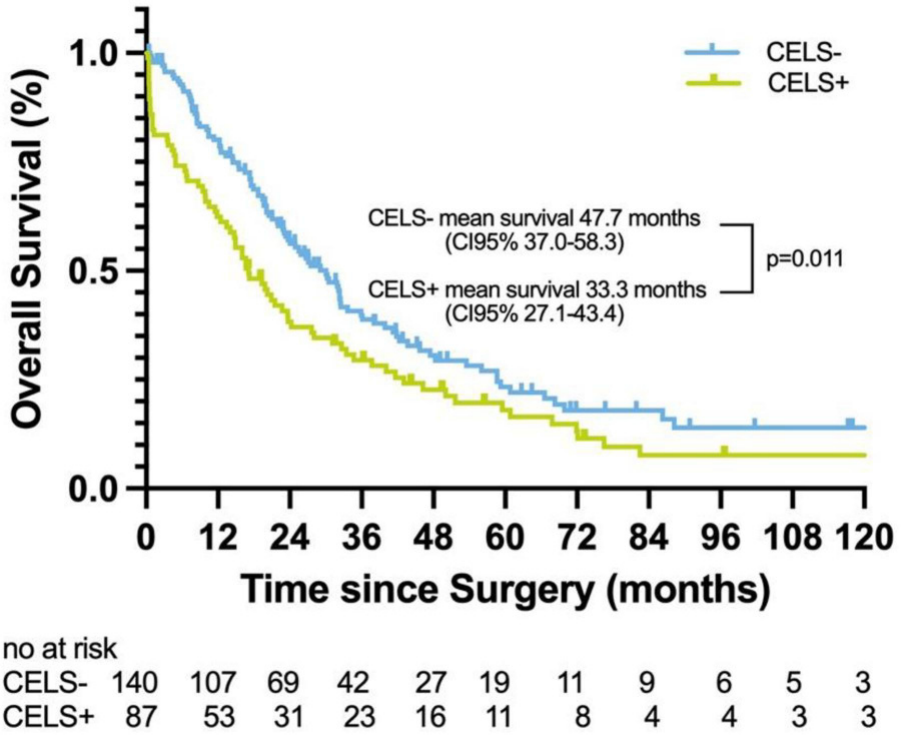
Kaplan–Meier analysis demonstrating overall survival comparing CELS-positive with CELS-negative patients.

Kaplan–Meier analysis was conducted to evaluate long-term survival outcomes. The median follow-up duration was 21.46 months. The mean survival estimate was 47.7 months in the CELS− group compared to 33.3 in the CELS+ group (*p* = 0.011). The sensitivity was 43.4%, the specificity was 78.8%, and the overall accuracy was 51.5%. The NPV was 29.3%, and the PPV was 87.4%. ROC analysis demonstrated low discriminatory ability (AUC, 0.61; *p* = 0.015) (Figure 4).

**Figure 5:**
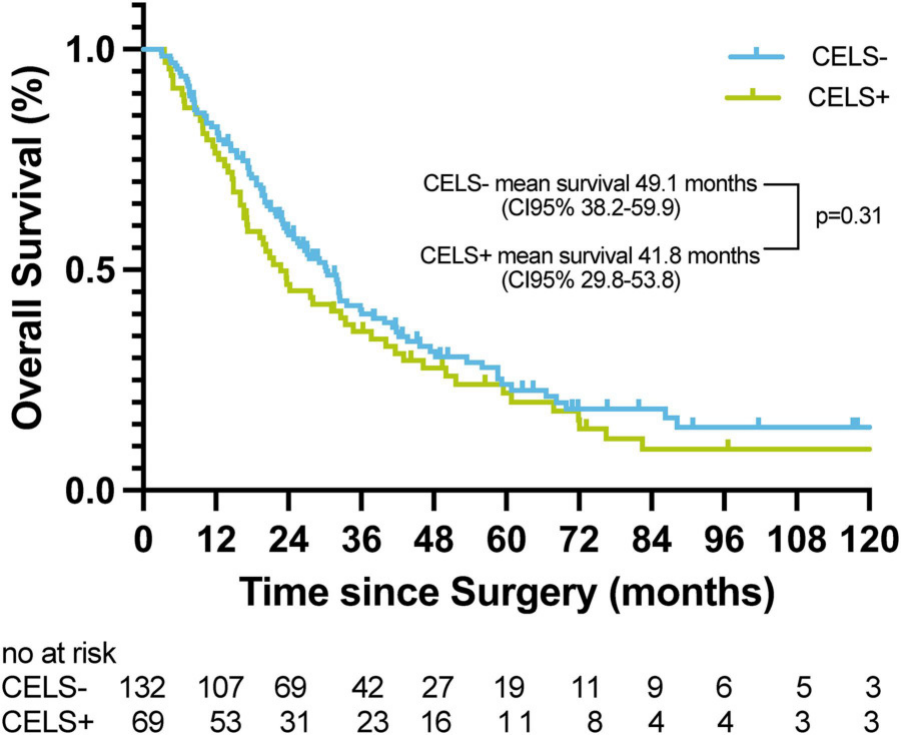
Adjusted Kaplan–Meier analysis demonstrating overall survival comparing CELS-positive with CELS-negative patients after excluding all patients with surgery-related death.

To account for confounding, SRD was excluded, leaving 205 cases for adjusted survival analysis. After exclusion, no significant mean survival differences was observed (CELS−: 49.1 months vs. CELS+: 41.8 months; *p* = 0.31). The adjusted predictive performance for long-term mortality showed a sensitivity of 37.9%, a specificity of 78.8%, and an overall accuracy of 48.3%; the NPV and PPV were 30.1% and 84.1%, respectively. After excluding SRD, ROC analysis demonstrated no discriminatory ability for long-term mortality (AUC = 0.58; *p* = 0.071) (Figure 5).

No significant differences in recurrence-free survival were identified (CELS−: 36.1 months vs. CELS+: 27.6; *p* = 0.19) (Supplementary Figure 1).

The sensitivity, specificity, and accuracy for disease recurrence were 36.4%, 58.6%, and 44.9% respectively. The NPV and PPV were 36.4% and 58.6%, respectively. ROC analysis demonstrated no discriminatory ability (AUC = 0.475; *p* = 0.53).

To further adjust for potential confounding, analyses were repeated after excluding patients with positive tumor resection margins (R1; *n* = 44). No significant mean difference in recurrence-free survival was observed between groups (CELS−: 38.1 months vs. CELS+: 30.2 months; *p* = 0.371) (Supplementary Figure 2). The predictive performance of CELS for RFS remained poor [sensitivity, 32.1%; specificity, 60.8%; accuracy, 43.7%; NPV, 37%, PPV, 54.7%; AUC = 0.46 (95% CI: 0.37–0.55); *p* = 0.41]. Overall, no relevant differences in oncological survival outcomes were observed between groups.

### Individual component analysis in the prediction of SRD

The individual components of the CELS were analyzed to assess their association with SRD, focusing on absolute risk increase and number needed to harm (NNH) (Table 3). Variables including PHH, PHLF, posthepatectomy bile leakage (PHBL), and intraoperative blood loss were dichotomized (event present vs. event not present). PHBL demonstrated a sensitivity of 45.5 and a specificity of 76.2%. The absolute risk increase for SRD was 10.1%, which corresponds to a number needed to harm of 10 patients.

**Table 3:** Individual component analysis including sensitivity, specificity, and accuracy as well as relative risk reduction to predict surgery-related death.

Component	Sensitivity (%)	Specificity (%)	Accuracy (%)	AUROC^a^	RRI^b^ (95% CI)	NNH^c^ (95% CI)
PHBL^d^	45.5	76.2	73.4	0.60	10.1% (−0.4–20.5)	10.0 (4.9–infinity)
PHH^e^	18.2	91.7	84.6	0.69	35.61 (15.6–55.6)	2.8 (1.8–6.4)
PHLF^f^	63.6	94.5	91.5	0.791	39.96 (20.31–59.6)	2.5 (1.7–4.9)
BL > 2 L^g^	50.0	91.4	85.5	0.55	10.3 (−6.9–27.5)	9.7 (3.6–infinity)
CELS^h^	81.8	66.3	67.8%	0.74	17.83 (8.8–26.8)	5.6 (3.7–11.3)

a Area under the receiver operating curve.

b Relative risk increase.

c Numbers needed to harm (numbers of patients needed to have a component endpoint event until the occurrence of surgery-related death).

d Posthepatectomy bile leakage.

e Posthepatectomy hemorrhage.

f Posthepatectomy.

g Blood loss exceeding 2 L.

h Composite endpoint for liver surgery.

Posthepatectomy hemorrhage was associated with a sensitivity of 63.6 and a specificity of 94.5% for predicting SRD. The relative risk increase for SRD in patients with PHH was 35.61%, resulting in an NNH of 2.8 patients.

PHLF was found to be the most effective predictor of SRD, with a sensitivity of 63.6% and a specificity of 94.5%, leading to a relative risk increase of 10.3% and an NNH of 2.5 patients.

Intraoperative blood loss exceeding 2 L was associated with a moderate sensitivity of 50% and specificity of 91.4%, corresponding to a moderate risk increase of 8.37% and a NNH of 12.0 patients.

In contrast, the combination of all endpoints resulting in CELS was associated with a moderate risk increase for SRD of 17.8% and an NNH of 5.6 patients.

## Discussion

We conducted a validation study to assess the effectiveness of a novel composite endpoint (CELS) in predicting surgery-related death in a cohort of patients with intrahepatic cholangiocarcinoma undergoing curative resection. To our knowledge, this represents the first validation of CELS outside of its original development cohort, in a clinically and geographically distinct population within a different healthcare system.

In our cohort, CELS demonstrated adequate predictive performance for SRD, with acceptable discriminatory ability (AUC = 0.74). Although LOS differed significantly between CELS-positive and CELS-negative patients, the predictive ability of CELS for prolonged LOS was moderate.

However, several methodological aspects should be discussed when interpreting this new composite endpoint for liver surgery.

The endpoint integrates multiple components with varying degrees of severity, resulting in substantial qualitative heterogeneity in their clinical relevance. This likely explains the high false-positive rate of CELS in predicting SRD (30.4%) observed in our cohort. For example, minor complications such as grade A bile leakage may increase postoperative morbidity but are unlikely to contribute to mortality (7, 8). Excluding lower-grade complications might improve the accuracy of the endpoint, albeit at the cost of larger required sample sizes. As emphasized by McCoy et al., disparities in the clinical importance of component events warrant caution when interpreting composite endpoints (9).

In an exploratory analysis (data not shown), we evaluated whether the predictive performance of CELS could be improved by modifying its component structure. We excluded PHBL from the composite. This adjustment resulted in a notable improvement in predictive accuracy, with the AUC increasing to 0.78. Although these findings suggest that refinement of the composite components may enhance clinical utility, further investigation in larger and more diverse cohorts is required to systematically evaluate and validate such modifications.

Furthermore, it should be discussed whether CELS is an appropriate endpoint for all patients undergoing all types of liver surgery or whether its application should be restricted to specific surgical subgroups. As reported in a meta-analysis by van Keeulen et al., the extent of hepatic resection, the presence or absence of biliary reconstruction, and the underlying tumor type are critical determinants of postoperative complication profiles and outcomes, with SRD rates reported to be as high as 11.4% (10). Our cohort mainly underwent extensive hepatic resections, frequently in combination with vascular or visceral reconstruction. This high-risk surgical profile is reflected in the elevated surgery-related mortality rate of 9.6% in the CELS cohort and 8.9% in the overall cohort. Liver resection for intrahepatic cholangiocarcinoma is associated with high rates of major complication (Clavien–Dindo >3a), as more extensive resections are often required (11–13). Given this complexity, a more tailored endpoint may be warranted to adequately capture the risk profile and clinically relevant outcomes in this subgroup.

Another issue concerns the general benefit of CELS as a surrogate for surgery-related death. Surgery-related death, defined as 90-day postoperative mortality, already constitutes a clinically meaningful and time-bound endpoint. Given a surgery-related mortality rate of 10.4% in Germany, this outcome alone may serve as a robust endpoint, as its high event rate is sufficient to require an acceptable sample size (13).

Instead of predicting short-term mortality, the development of an effective composite endpoint to predict long-term survival would arguably be of greater academic and clinical relevance. As stated by Pawlik et al., CELS was associated with poorer overall survival in the internal validation cohort, suggesting a possible underlying link between postoperative complications and cancer progression. Several authors have proposed that many tumors may establish clinically undetectable micrometastases at local or distant sites prior to resection. These micrometastases may remain in a state of dormant equilibrium between cellular proliferation and apoptosis. However, systemic inflammatory responses and local tissue trauma can unpredictably unleash their potential for growth (14, 15). In this context, a potential association between CELS and overall and recurrence-free survival appears logical. However, in our cohort, CELS was not associated with decreased overall or recurrence-free survival after appropriate adjustment. This finding may be explained by the recurrence pattern of iCCA, which predominantly recurs intrahepatically (53.2%) rather than through circulating micrometastases at distant sites (16). However, this remains a speculative hypothesis.

An additional conceptual limitation relates to the design of CELS as a surrogate predictor of SRD, rather than including SRD as a component of the composite itself. As emphasized in prior methodological frameworks, the interpretability of composite outcomes is improved when all components are of similar clinical significance and trend in the same direction. In our cohort, the relative risk increases associated with the individual CELS components varied markedly, underscoring the unequal contribution of each element to the prediction of SRD. This heterogeneity complicates interpretation and may weaken the construct validity of the composite endpoint.

To address these limitations, we propose that future work should consider the development of a composite endpoint, such as a major events in liver surgery metric, for the evaluation of surgical techniques. Such an endpoint could include surgery-related death, grade B and C posthepatectomy liver failure, and other well-established adverse events associated with poor outcomes in liver surgery. Comparable outcome parameters that facilitate the conduct of clinical trials in liver surgery by improving statistical efficiency and comparability of evidence are urgently needed.

In summary, the new composite endpoint for liver surgery, consisting of posthepatectomy bile leakage, liver failure, hemorrhage, and intraoperative blood loss exceeding 2 L, demonstrated sufficient discriminatory ability for predicting surgery-related mortality in our external cohort. Although CELS shows promise, further refinement is required to ensure that clinical relevance, rather than statistical efficiency alone, guides the design and adoption of composite endpoints for liver surgery.

### Limitations

This study has several limitations that should be acknowledged. Most notably, its retrospective design may introduce inherent biases, such as selection and information bias, which could impact the validity of our findings. However, as CELS has not previously been evaluated outside its original development cohort, the primary aim of our study was to assess its predictive performance in an independent retrospective cohort before considering its application in prospective trials or its use in clinical decision-making.

Only patients with complete data across all relevant variables were included, potentially leading to an overestimation of surgery-related mortality. This bias arises from because nearly all patients who experienced SRD were captured in the final analysis, as their clinical data were comprehensively documented during the index hospitalization up to the time of death. In contrast, patients who survived but had incomplete follow-up data were excluded due to missing variables. To address this imbalance and avoid a skewed representation of SRD, we ensured that SRD data were included in both the CELS analysis and the overall cohort evaluation. This approach was intended to preserve the accuracy of mortality estimates and support an unbiased assessment of CELS performance in predicting SRD.

Moreover, while the sample size may appear limited, this was a deliberate methodological choice. CELS was originally developed to improve the feasibility of clinical trials by enabling meaningful analyses in smaller patient populations. Therefore, our use of a modestly sized cohort aligns with the intended application of the endpoint and allows assessment of whether its purported statistical efficiency translates into practical utility.

## Data Availability

The raw data supporting the conclusions of this article will be made available by the authors, without undue reservation.
