# Supplementary material for: A novel endpoint for liver surgery predicts surgery-related mortality: external validation in a surgical cohort with intrahepatic cholangiocarcinoma
Source: Front Surg. 2026 May 15;13:1835401. doi: 10.3389/fsurg.2026.1835401 (PMC13218855; doi:10.3389/fsurg.2026.1835401)
Supplement: Supplementary file 1 [file Datasheet1.docx]

Supplementary Material

**A.**

Table 1a: Missing data analysis.

| **Missing data variables** | **(n=41)** |
| --- | --- |
| **ASA** | 1 |
| **PHBL** | 1 |
| **PHH** | 1 |
| **PHLF** | 1 |
| **CD ≥ 3a** | 4 |
| **Intraoperative blood loss** | 20 |
| **T-stage** | 2 |
| **Ca 19-9 U/l** | 11 |

**B.**


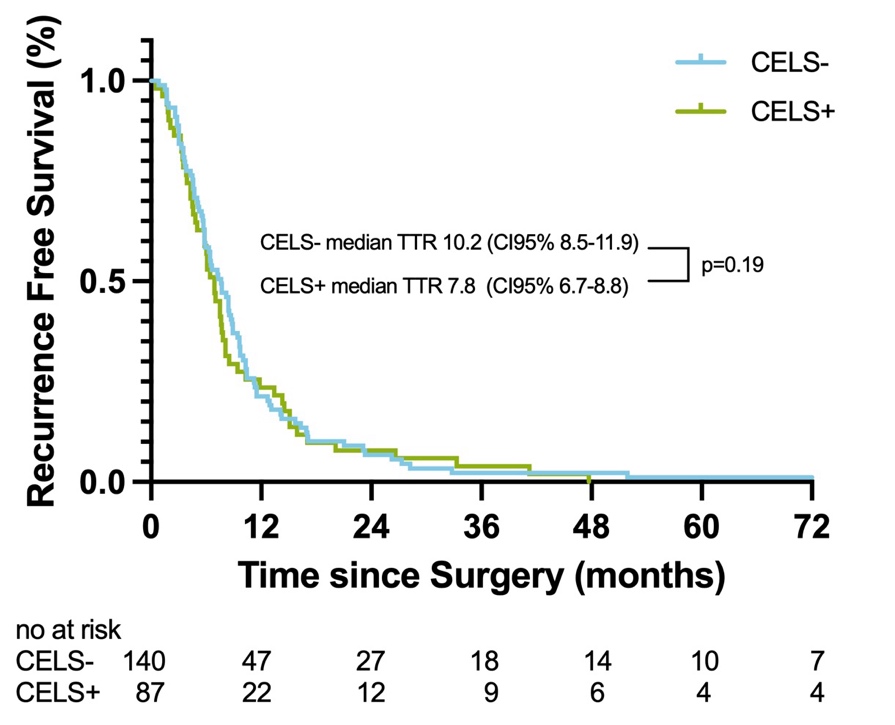


Supplementary Figure 1: Kaplan Meier Analysis demonstrating recurrence free survival comparing CELS positive with CELS negative patients

**C.**


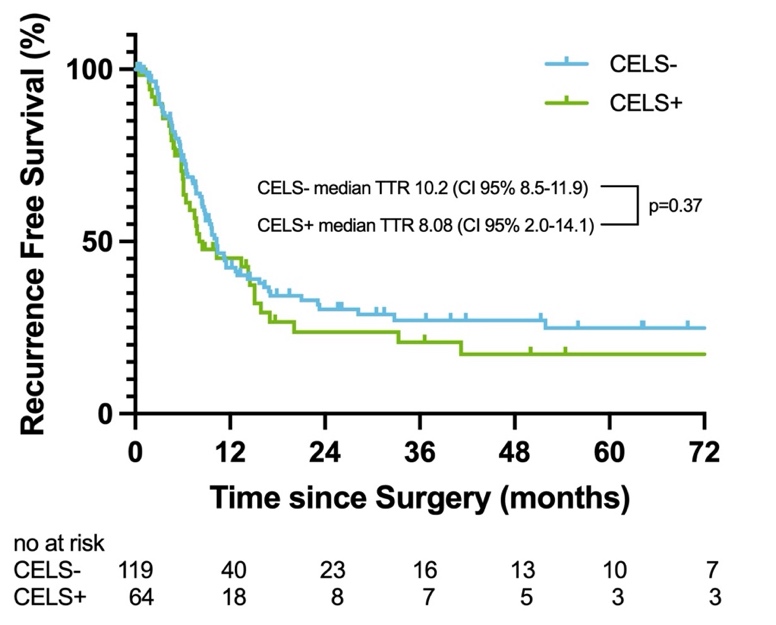


Supplementary Figure 2: Kaplan Meier Analysis demonstrating recurrence free survival comparing CELS positive with CELS negative patients after excluding R1-resections
